# Establishment of CRISPR/Cas9 Genome-Editing System Based on Dual sgRNAs in *Flammulina filiformis*

**DOI:** 10.3390/jof8070693

**Published:** 2022-06-30

**Authors:** Xiaotian Liu, Jianghan Dong, Jian Liao, Li Tian, Hao Qiu, Tao Wu, Feng Ge, Jing Zhu, Liang Shi, Ailiang Jiang, Hanshou Yu, Mingwen Zhao, Ang Ren

**Affiliations:** 1 Key Laboratory of Microbiological Engineering of Agricultural Environment, Ministry of Agriculture, Department of Microbiology, College of Life Sciences, Nanjing Agricultural University, Nanjing 210095, China; sheltonliu@foxmail.com (X.L.); 10318127@njau.edu.cn (J.D.); 10317128@njau.edu.cn (J.L.); 2020116058@stu.njau.edu.cn (L.T.); 2020816130@stu.njau.edu.cn (H.Q.); wt1170450810@hotmail.com (T.W.); 2021816129@stu.njau.edu.cn (F.G.); jingzhu@njau.edu.cn (J.Z.); shiliang@njau.edu.cn (L.S.); aljiang@njau.edu.cn (A.J.); yuhans@njau.edu.cn (H.Y.); mwzhao@njau.edu.cn (M.Z.); 2 Sanya Institute of Nanjing Agricultural University, Sanya 572025, China; 3 Institute of Biology, Guizhou Academy of Sciences, Guiyang 550009, China

**Keywords:** *Flammulina filiformis*, CRISPR/Cas9, gene editing, dual sgRNAs, U6 promoter, *pyrG*

## Abstract

*Flammulina filiformis*, previously known as Asian *Flammulina velutipes*, is one of the most commercially important edible fungi, with nutritional value and medicinal properties worldwide. However, precision genome editing using CRISPR/Cas9, which is a revolutionary technology and provides a powerful tool for molecular breeding, has not been established in *F. filiformis*. Here, plasmids harboring expression cassettes of Basidiomycete codon-optimized Cas9 and dual sgRNAs targeting *pyrG* under the control of the *gpd* promoter and FfU6 promoter, respectively, were delivered into protoplasts of *F. filiformis* Dan3 strain through PEG-mediated transformation. The results showed that an efficient native U6 promoter of *F. filiformis* was identified, and ultimately several *pyrG* mutants exhibiting 5-fluorooric acid (5-FOA) resistance were obtained. Additionally, diagnostic PCR followed by Sanger sequencing revealed that fragment deletion between the two sgRNA target sites or small insertions and deletions (indels) were introduced in these *pyrG* mutants through the nonhomologous end joining (NHEJ) pathway, resulting in heritable changes in genomic information. Taken together, this is the first report in which a successful CRISPR/Cas9 genome-editing system based on dual sgRNAs was established in *F. filiformis*, which broadens the application of this advanced tool in Basidiomycetes.

## 1. Introduction

As one of the most common edible mushrooms worldwide, *Flammulina filiformis*, also named winter mushroom or enokitake, and previously known as *Flammulina velutipes*, has been renamed according to a recent report [1]. Based on phylogenetic analyses, genetic structure analyses, and haplotype network analysis, Wang et al. proposed that *F. filiformis* cultured in East Asia should be treated as a separate species that is different from European *F. velutipes*. It is widely recognized that *F. filiformis* is rich in amino acids, vitamins, minerals, and unsaturated fatty acids, which are essential and beneficial to human health [2]. In addition to its nutritional value, an increasing amount of research has focused on the medicinal properties of *F. filiformis*, such as immune-modulatory, anti-inflammatory, antioxidant, and intestinal flora activities [3,4,5,6]. With the development of the *F. filiformis* industry, generating highly valuable cultivars and improving the quality have become two important issues faced in *F. filiformis* industrial production. Unfortunately, classical breeding is time-consuming and laborious due to the tedious crossing steps [7]. In contrast, molecular breeding has attracted increasing attention, largely due to its flexibility, accuracy, and high efficiency [8,9,10].

CRISPR/Cas9-mediated genome editing is a revolutionary technology that has been widely applied in filamentous fungi, including Basidiomycetes [11,12,13]. However, due to the limited genetic manipulation and transformation methods, there have been relatively few reports describing successful CRISPR systems in edible fungi. For the convenience of screening and verification, most studies attempting to establish CRISPR systems in Basidiomycetes firstly preferred to choose genes that encoded clear morphological phenotypes or physiological properties for editing, such as *pyrG* in *Pleurotus eryngii* [14] and *ura3* in *Ganoderma lucidum* [15]. Because *pyrG*/*ura3* encodes orotidine-5′-monophosphate decarboxylase, which is not only involved in the key pathway of uracil synthesis, but also can convert 5-fluorooric acid (5-FOA) into the toxic substance 5-fluorouridine, *pyrG*/*ura3* mutants survive and wild-type (WT) die on a medium supplied with uracil and 5-FOA.

Efficient expression of Cas9 and sgRNA (single-guide RNA) guarantees successful CRISPR genome editing. DNA double-strand breaks (DSBs) caused by Cas9 can be repaired mainly via nonhomologous end joining (NHEJ) or homology-directed repair (HDR) mechanisms [16], which alter genetic information near the target site permanently. Theoretically, once the genomic information of CRISPR-targeted recipient cells has been changed, there will be no need for the existence of Cas9 and sgRNA. However, given that there are few reports on inducible promoters in Basidiomycetes, the conditional expression of Cas9 and sgRNA has not been feasible yet. Thus, the RNA polymerase (Pol) II constitutive promoters are adopted to express Cas9, while Pol III promoters such as U6 are suitable for expression of sgRNA, owing to the fact that mRNA processing events such as splicing, 5′ capping, and 3′ poly(A)-tail addition are not involved. Up to now, the *gpd* (glyceraldehyde-3-phosphate dehydrogenase) or *ef3* (elongation factor 3) promoter (Pol II) and U6 promoter (Pol III) have been successfully applied in some edible fungi, including *P. eryngii* [14], *Pleurotus ostreatus* [17], and *Lentinula edodes* [18].

In essence, checking the integrity of genomic information is the core work of screening and verification of mutants after gene editing. Several strategies have been developed to track changes in sequence information, such as Sanger sequencing and next-generation sequencing (NGS) [19], despite the fact that they are time-consuming and expensive. The PCR/restriction enzyme (PCR/RE) method [20], which is a sequencing-free manner, depends on electrophoresis by means of visualizing the differences in PCR products between the WT and mutants. Unfortunately, small insertions and deletions (indels) are the most common mutation types based on a single sgRNA in the CRISPR/Cas9 system through NHEJ [21], which means sequence differences cannot be distinguished using electrophoresis. The dual-sgRNA-mediated fragment-deletion strategy has attracted increasing interest [22,23,24,25], probably due to its high efficiency and divergence visualization. In this method, two independent DSBs are simultaneously generated at the two sgRNA-targeted loci, and then the DSBs rejoin through NHEJ, resulting in the generation of chromosomal segment deletion between the two cutting sites of Cas9.

Although many attempts have been made [26,27], so far there is no report on successful CRISPR-mediated gene editing in *Flammulina*. In the present study, by identifying U6 promoters in the *F. filiformis* genome, we obtained *pyrG* mutants with fragment or base deletion profiting from expression of Basidiomycete codon-optimized Cas9 and dual sgRNAs under the control of the *gpd* promoter and FfU6 promoter, respectively.

## 2. Materials and Methods

### 2.1. Strains and Culture Conditions

The *F. filiformis* strain Dan3, obtained from Shanghai Academy of Agricultural Sciences (SAAS), was used as the host for gene disruption. The *F. filiformis* was cultured in CYM medium (10 g/L maltose, 20 g/L glucose, 2 g/L tryptone, 2 g/L yeast extract, 0.5 g/L MgSO_4_·7H_2_O, and 4.6 g/L KH_2_PO_4_) at 25 °C. Rescreening of possible 5-FOA-resistance transformants was performed through culturing on MM medium (20 g/L glucose, 2 g/L L-asparagine, 0.5 g/L MgSO_4_·7H_2_O, 0.46 g/L KH_2_PO_4_, and 1 g/L K_2_HPO_4_) containing 600 µg/mL 5-FOA (Sangon Biotech, Shanghai, China) and 100 µg/mL uracil (Sangon Biotech). The *Escherichia coli* strain DH5α was used for vector construction.

### 2.2. Selection of sgRNAs of pyrG and U6 Promoters of F. filiformis

To design the sgRNA sequence used in gene editing, the nucleotide sequence of *pyrG* was uploaded to the online design tool EuPaGD (http://grna.ctegd.uga.edu (accessed on 9 November 2021)). Only those with high scores and “G” at 5′ were chosen as candidate sgRNAs.

In order to identify the FfU6 promoters, we performed a BLAST search of the *F. filiformis* genome using the nucleotide sequence of human U6 snRNA (NCBI accession: NR_004394.1) as a query to search for its homologs by means of BioEdit software, and then selected 400 bp upstream of the transcription start site (TSS) as the predicted FfU6 promoters.

### 2.3. In Vitro Cas9 Cleavage Assay

The primer pairs FfpyrG-vitro-F1/FfpyrG-vitro-R1 and FfpyrG-vitro-F2/FfpyrG-vitro-R2 (Table 1) were used to amplify the *pyrG* fragments containing the respective target site. First, 5 µL Cas9 nuclease (500 ng/µL) and 10 µL sgRNA (50 ng/µL) purchased from GenScript (Nanjing, China) were incubated for 5 min at 37 °C to form a ribonucleoprotein (RNP) complex. Subsequently, the RNP complex was incubated with the PCR-amplified fragment (100 ng) in Cas9 reaction buffer for 1 h at 37 °C. Finally, the mixture was visualized using 1.5% agarose gel electrophoresis.

### 2.4. Construction of CRISPR Plasmids

Plasmids used for *pyrG* editing were derived from the vector pXT-300, which successfully enabled genome editing in *G. lucidum* (data not shown), harboring the *gpd* promoter and Basidiomycete codon-optimized Cas9. Cas9 was optimized according to humanized *Streptococcus pyogenes* Cas9 (SpCas9) open reading frame (ORF) in the commercial vector pX330 (addgene #42230), with a SV40 NLS (nuclear localization signal of simian virus 40) and a nucleoplasmin NLS located at 5′ and 3′, respectively. For the construction of the sgRNA expression cassette, the primer pair FfU6-1-F1/FfU6-1-R1 (Table 1) was used to amplify the FfU6-1-1 promoter from the *F. filiformis* genomic DNA. Using the primer pair pyrG-sgRNA1-F/pyrG-sgRNA1-R, the fragment sgRNA1 containing FfpyrG-sgRNA1, backbone region of gRNA scaffold, and a “TTTTTT” transcription terminator were amplified from pX330. Similarly, fragments FfU6-1-2 and sgRNA2 were amplified by the primer pairs FfU6-1-F2/FfU6-1-R2 and pyrG-sgRNA2-F/pyrG-sgRNA2-R, respectively. By means of a ClonExpress Ultra One Step Cloning Kit (Vazyme, Nanjing, China), the four purified fragments—FfU6-1-1, sgRNA1, FfU6-1-2, and sgRNA2—were sequentially ligated into the *Not* I digested vector pXT-300, yielding plasmid pXT-302-FfpyrG-1. The plasmid-containing FfU6-3 promoter, named pXT-302-FfpyrG-3, was constructed through the same steps as described above, except for the primers used. Briefly, the primer pairs FfU6-3-F1/FfU6-3-R1 and FfU6-3-F2/FfU6-3-R2 were used to amplify the fragments FfU6-3-1 and FfU6-3-2, respectively. Subsequently, FfU6-3-1, sgRNA1, FfU6-3-2, and sgRNA2 were ligated into the *Not* I digested vector pXT-300, yielding plasmid pXT-302-FfpyrG-3.

### 2.5. PEG-Mediated Transformation of Protoplasts

Protoplast preparation and transformation were performed as described previously [15] with minor modifications. The mycelia were collected and washed with 0.6 M mannitol (Sangon Biotech) and digested with 2% (*w*/*v*) lywallzyme (Guangdong Institute of Microbiology, Guangzhou, China) for 3 h at 30 °C. After filtering insufficiently digested mycelia, the protoplasts were resuspended in MTC buffer (0.6 M mannitol, 100 mM CaCl_2_, and 100 mM Tris-HCl at pH 7.5) and adjusted to a final concentration of approximately 10^7^ cells/mL. Then, the protoplasts in 160 μL MTC buffer were transformed with 20 μg linearized pXT-302-FfpyrG-1/3 plasmid, 10 μL 20 mM aurintricarboxylic acid (ATA) (Sigma-Aldrich, Shanghai, China), 5 μL 50 mM spermidine (Sigma-Aldrich), 2 μL 50 mg/mL heparin (Sangon Biotech), and 60 μL PTC buffer (40% polyethylene glycol (PEG) 3350, 100 mM CaCl_2_, and 10 mM Tris-HCl at pH 7.5). Subsequently, the mixture was incubated on ice for 30 min, then 1 mL PTC buffer was added and incubated for another 30 min at 25 °C. After centrifugation at 4000 rpm for 10 min, protoplasts were resuspended in 1 mL liquid CYM medium containing 0.6 M mannitol for 24–30 h at 25 °C.

### 2.6. Screening and Verification of Transformants

Protoplasts resuspended in 1 mL liquid CYM medium were poured onto CYM selective regeneration medium containing 0.6 M mannitol and 600 µg/mL 5-FOA. A total of 28 days after the PEG-mediated protoplast transformation, all transformants present on CYM selective regeneration medium were transferred to MM medium containing 600 µg/mL 5-FOA and 100 µg/mL uracil. After 14 days of culture, with the use of MightyAmp^TM^ DNA Polymerase Ver.3 (Takara, Beijing, China), all isolates were verified using diagnostic PCR or sequencing analysis using mycelia as the template. With the primers FfpyrG-chk-F/FfpyrG-chk-R, PCR amplification of *pyrG* fragments of all transformants that contained the target sites were compared with the WT *pyrG* sequence using Sanger sequencing to determine whether indels occurred at or near the expected sites.

## 3. Results

### 3.1. In Vitro Cas9 Cleavage Assay

**Figure 1:**
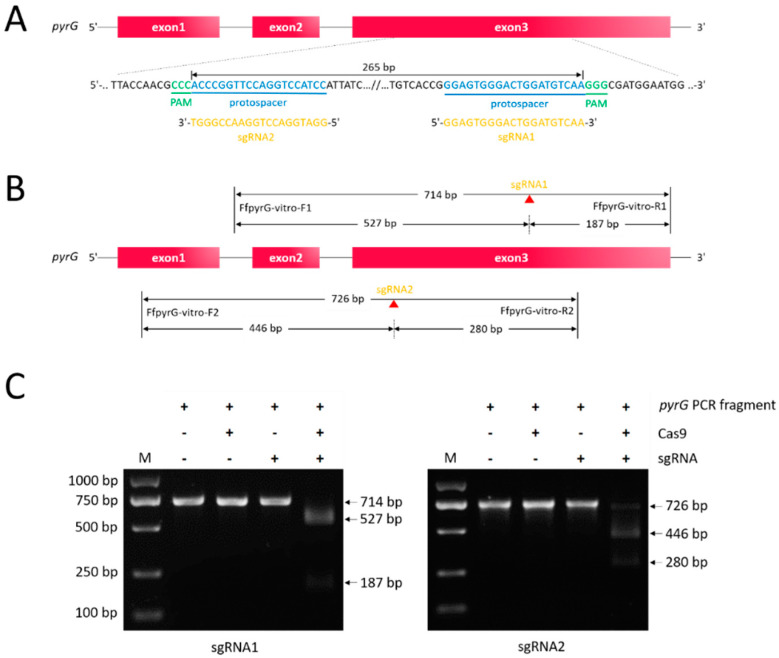
**Schematic illustration of sequence information and in vitro Cas9 cleavage assay.** (**A**) Sequences of the two sgRNAs targeting *pyrG* are shown in yellow font, both of which were located at exon3. Sequence directions are all 5′–3′ as shown. Schematic representation of exons was not drawn to scale. (**B**) Fragments required for in vitro Cas9 cleavage assay were amplified with primer pairs FfpyrG-vitro-F1/FfpyrG-vitro-R1 and FfpyrG-vitro-F2/FfpyrG-vitro-R2, respectively. Target regions that could be recognized by sgRNA1 or sgRNA2 were cleaved by Cas9 (red triangles), yielding two small fragments. (**C**) Visualization of the in vitro Cas9 cleavage assay through agarose gel electrophoresis. M, marker.

Successful genome editing was ensured by high-efficiency sgRNA. To verify whether the *pyrG* target sites could be recognized and cleaved by Cas9 endonuclease under the guidance of designed sgRNAs (Figure 1A, sgRNA1: 5′-GGAGTGGGACTGGATGTCAA-3′; sgRNA2: 5′-GGATGGACCTGGAACCGGGT-3′), we performed a cleavage validation experiment in vitro. Using genomic DNA of *F. filiformis* as the template, the *pyrG* fragments containing target regions with sizes of 714 bp and 726 bp were amplified through the primers FfpyrG-vitro-F1/FfpyrG-vitro-R1 and FfpyrG-vitro-F2/FfpyrG-vitro-R2, respectively. Theoretically, cleavage of the 714 bp fragment caused by RNP complex would yield two small bands of 527 bp and 187 bp, while cleavage of the 726 bp fragment would yield two small bands of 446 bp and 280 bp (Figure 1B). As expected, under the circumstance that only Cas9 or sgRNA was present in the reaction system, there was no observed difference in fragments compared with the groups to which only *pyrG* PCR fragments were added. On the contrary, the 714 bp and 726 bp PCR fragments were almost completely digested and two respective small bands appeared, provided both Cas9 and sgRNA existed in the reaction system (Figure 1C). The results were consistent with the theory, which indicated that the targeting efficiency of the two sgRNAs was high enough, and they could be utilized in subsequent experiments.

### 3.2. Four U6 Promoters of F. filiformis Were Identified Based on Homologous Search

**Figure 2:**
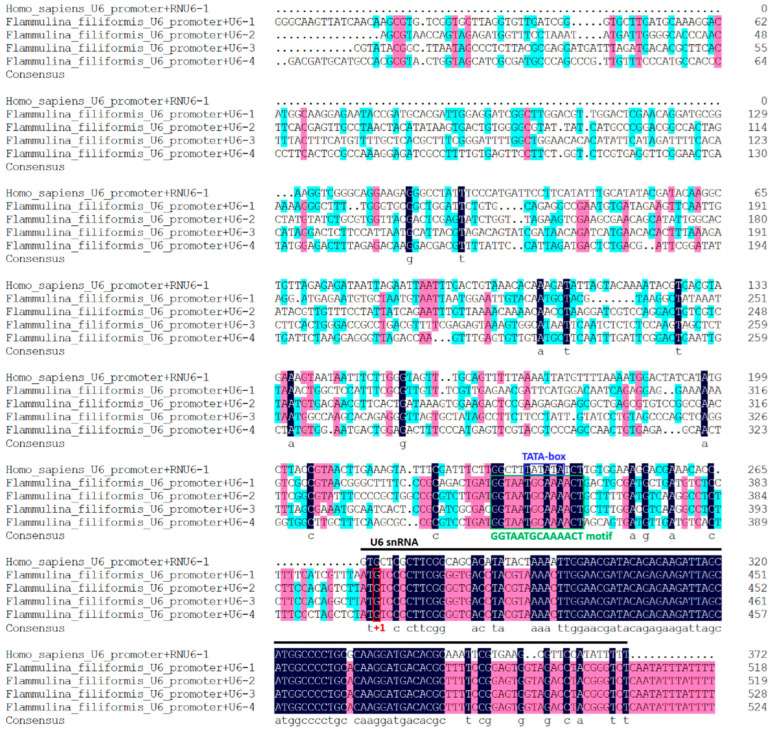
**Analysis of *F. filiformis* U6 and promoter sequences.** Multiple alignments of *F. filiformis* and human U6 snRNA are illustrated. Black lines indicate human U6 snRNA sequence. The conserved elements TATA box and “GGTAATGCAAAACT” motif are indicated by blue and green frames, respectively. The nucleotides “G” that could be recognized by FfU6 promoters for transcription initiation (+1) are labeled with a red box. Different colors denote different levels of sequence identity. The strictly conserved nucleotides (100% identity) are highlighted in black, while those with ≥75% and ≥50% identity are highlighted in red and blue, respectively. The multiple sequence alignments were performed using DNAMAN software.

Four candidate genes, termed FfU6-1, FfU6-2, FfU6-3, and FfU6-4, were identified through a homologous search in the *F. filiformis* genome with the human U6 snRNA gene, and all of them shared a conserved region with RNU6-1. Since nucleotide “G” was the TSS of U6 promoters [28], we selected 400 bp upstream of the TSS as the predicted FfU6 promoters. An analysis of multiple sequence alignments between FfU6 promoters and *Homo sapiens* U6 promoter showed that the promoter regions of these U6 varied greatly, including the TATA-like box, which was critical for transcription in the Pol III promoter, according to previous reports [29] (Figure 2). Interestingly, the four FfU6 promoters lacked a typical TATA box but contained a “GGTAATGCAAAACT” motif, which could be beneficial to effective transcription. In view of this, FfU6-1 and FfU6-3 promoters were randomly selected and adopted for the respective expression of sgRNA.

### 3.3. Transformants Harboring pyrG Fragment Deletion or Small Indels Were Obtained

**Figure 3:**
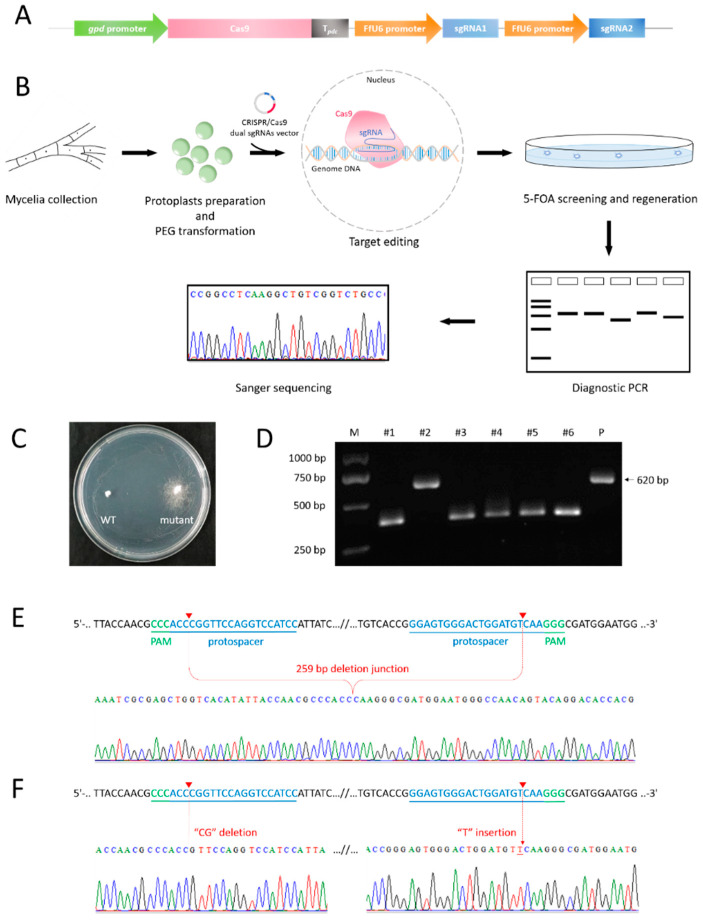
**Screening and confirmation of *pyrG* mutants of *F. filiformis*.** (**A**) Schematic diagram of vectors used in mutation of *pyrG*. (**B**) PEG-mediated protoplast transformation and screening process. (**C**) Rescreening on MM medium containing 5-FOA and uracil. (**D**) Diagnostic PCR of *pyrG* fragment harboring target sites. “P” indicates positive amplification control amplified from WT, with a size of 620 bp. (**E**) Sequencing result confirming the *pyrG* mutants harboring fragment deletion. (**F**) Sequencing result of the *pyrG* mutant harboring small indels.

Twenty-eight days after the PEG-mediated protoplast transformation (Figure 3A,B), colonies with white and dense hyphae were selected from 5-FOA-containing CYM regeneration medium and transferred to MM medium supplied with 5-FOA and uracil. A total of six transformants that exhibited resistance to 5-FOA (Figure 3C) were obtained from several replicates, and all of them were subjected to PCR to amplify the *pyrG* fragment using mycelia as the template with the primer pair FfpyrG-chk-F/FfpyrG-chk-R (Figure 3D). The agarose gel electrophoresis result showed the presence of variant bands that were about 250 bp smaller than that of WT (positive amplification control, Figure 3E). After gel electrophoresis purification and recovery, PCR products were subjected to Sanger sequencing with the primer FfpyrG-chk-F or cloned into the pMD19-T vector for further sequencing with the universal primer M13F-47. The sequencing results showed that a total length of 259 bp in *F. filiformis pyrG* locus was deleted, which was the interval between dual sgRNAs; more specifically, a region starting from 3 bp upstream of the PAM (the reverse complement of CCC; i.e., GGG) of sgRNA2 and ending at 3 bp upstream of the PAM (GGG) of sgRNA1 (Figure 3F). However, the molecular mechanism by which two DSBs generated by Cas9 directly rejoin together to generate chromosomal fragment deletion in vivo remains unclear [24]. In addition, one mutant harboring small indels (“CG” deletion and “T” insertion), whose size of PCR product was almost the same as that of WT, was determined (Figure 3F). Notably, all mutants obtained in the present study benefited from FfU6-1 under current experimental conditions. Taken together, our results demonstrated that the dual-sgRNA system could be applied as an efficient tool to introduce fragment deletion or indels for CRISPR/Cas9-mediated genome editing in *F. filiformis*.

## 4. Discussion

Since the first application in eukaryotic cells about a decade ago [16,28], CRISPR/Cas9 has exhibited its huge potential with great success in a tremendous number of species, strongly promoting the development of biotechnology, agriculture, and medicine [30]. However, in view of numerous species of Basidiomycetes, establishment of CRISPR/Cas9 system has just begun and remains scarce, especially in mushrooms, notwithstanding versatile and convenient application. Generally, determining appropriate target sites could be helpful for evaluation of editing efficiency, such as *pksP* and *lae1* in *Aspergillus fumigatus* [31] and *Trichoderma reesei* [32], respectively. Pigment production is blocked due to inactivation of these genes, which makes it easy to distinguish mutants from WT. As a negative selection marker, mutation of *pyrG*/*ura3* confers 5-FOA resistance in a host; meanwhile, the uracil synthesis pathway is interrupted. Here, we achieved the goal of fragment or base deletion of *pyrG* in *F. filiformis* by means of endogenous expression of Cas9 and dual sgRNAs.

According to the mechanism of CRISPR/Cas9, just a single sgRNA would be competent for gene disruption, despite the fact that dual sgRNAs were applied in this study. In practice, adoption of a dual-sgRNA strategy has obvious merits. First, a low mutation efficiency caused by a single sgRNA itself can be avoided as far as possible. Second, fragment deletion mediated by dual sgRNAs can be visualized directly via electrophoresis, which greatly improves the efficiency of mutant screening. Third, due to fragment deletion of the target region between the two sgRNAs, it is clear that a more complete deletion in terms of the primary structure is obtained in this manner, which is especially helpful in research on noncoding RNAs (ncRNAs) or other gene structures with biological functions [33,34]. Previous studies have reported several strategies for the expression of multiple sgRNAs; for instance, Pol III promoter::sgRNA tandem repeats [35], the endogenous tRNA-processing system [24], Csy4 ribonuclease [36], RZ ribozyme [37], hepatitis delta virus (HDV) and hammerhead (HH) ribozymes [38], and so on. An off-target effect that accompanies more sgRNAs, nevertheless, should be noticed.

CRISPR-related components and their delivery strategy will depend on the assay and cell type. In general, direct delivery of an in vitro Cas9/sgRNA complex (namely ribonucleoprotein (RNP)) or vectors containing Cas9-encoding and sgRNA-encoding information are the major methods [33]. Even though the Pol II promoter and Cas9 applied in this study were derived from *G. lucidum*, we still gained expected *pyrG* mutants. However, only the U6 promoter of *F. filiformis* could successfully screen mutants, while the use of the U6 promoter of *G. lucidum* failed (data not shown), probably due to unsuccessful transcription of sgRNA. Although a few articles using heterologous promoters reported successful CRISPR/Cas9 genome editing [17], it seems that most studies regarding CRISPR/Cas9 in Basidiomycete fungi, especially edible and medicinal fungi, tended to adopt their native promoters [14,39,40]. Furthermore, we noticed that the U6 promoter of *F. filiformis* did not contain a TATA box, and a similar phenomenon was also found in *G. lucidum* [39], despite the fact that it was vital for U6 transcription in many species [29]. We tried to analyze the U6 promoters in *P. eryngii*, and the same result was found (data not shown). Whether the lack of a TATA box is a common feature in edible and medicinal fungi remains to be further investigated. As classic genetic-manipulation methods, PEG-mediated transformation and *Agrobacterium tumefaciens*-mediated transformation were widely applied in filamentous fungi, generally, resulting in homologous or heterologous integration of CRISPR-related elements into the host genome. In this study, although we obtained *pyrG* mutants with fragment deletion or small indels via PEG-mediated transformation, the screening efficiency was relatively low. One reason could be that the vectors containing CRISPR-related elements were not delivered efficiently. There are few reports on autonomous replicators, except for AMA1 [41,42], which is only found in Aspergillus. Hence, CRISPR-related components will duplicate along with genome duplication. That is to say, the integration in genome is permanent, which does not cater to agriculture and food safety. On the contrary, an RNP complex formed by Cas9 and sgRNA, which are both transcribed and assembled in vitro; i.e., without restrictions on species, is an ideal strategy for genome editing due to its short half-life [32,43,44].

In conclusion, this was the first report to successfully establish a CRISPR/Cas9 genome-editing system through identifying native U6 promoters and applying a dual-sgRNA strategy in the edible mushroom *F. filiformis*, which broadened the application of this powerful tool in Basidiomycetes. Further research should be focused on increasing editing efficiency and targeting other genomic regions such as genes and functional elements.

## Figures and Tables

**Table 1:** List of oligonucleotides used in this study.

Name	Sequence (5′–3′)	Descriptions
FfpyrG-vitro-F1	ATGGCACATATCCTCAACTTTG	Amplification of *pyrG* for in vitro cleavage assay
FfpyrG-vitro-R1	TCATGCTGTTCTCTCCAAGTATG	
FfpyrG-vitro-F2	ACGCCGCTCGCGCCTCAAAACATTC	Amplification of *pyrG* for in vitro cleavage assay
FfpyrG-vitro-R2	TGGCCCATTCCATCGCCCTTGAC	
FfU6-1-F1	TCGGGAAGAGCAGAGCGGGCAAGTTATCAACAAGCGTG	Amplification of FfU6-1 promoter
FfU6-1-R1	TTGACATCCAGTCCCACTCCATTAAACGATGAAAAGGAGACATC	
pyrG-sgRNA1-F	GGAGTGGGACTGGATGTCAAGTTTTAGAGCTAGAAATAGCAAG	Amplification of sgRNA1
pyrG-sgRNA1-R	TCTAAAACAAAAAAGCACCGACTCGGTG	
FfU6-1-F2	GTCGGTGCTTTTTTGTTTTAGAGGGCAAGTTATCAACAAGCGTG	Amplification of FfU6-1 promoter
FfU6-1-R2	ACCCGGTTCCAGGTCCATCCATTAAACGATGAAAAGGAGACATC	
pyrG-sgRNA2-F	GGATGGACCTGGAACCGGGTGTTTTAGAGCTAGAAATAGCAAG	Amplification of sgRNA2
pyrG-sgRNA2-R	GGATCCTCTAGAGATGCGGCCGCTCTAAAACAAAAAAGCACCGAC	
FfU6-3-F1	TCGGGAAGAGCAGAGCGTATACGGCTTAATAGCCCTCTT	Amplification of FfU6-3 promoter
FfU6-3-R1	TTGACATCCAGTCCCACTCCATAAGCCTGTGGAAGAGAGGC	
FfU6-3-F2	GTCGGTGCTTTTTTGTTTTAGACGTATACGGCTTAATAGCCCTCTT	Amplification of FfU6-3 promoter
FfU6-3-R2	ACCCGGTTCCAGGTCCATCCATAAGCCTGTGGAAGAGAGGC	
FfpyrG-chk-F	CAACTTTGCGAGCATAGACCC	Amplification of *pyrG* for sequencing
FfpyrG-chk-R	AATAATCCGTCCCATACACCC	

## Data Availability

Not applicable.
